# Uterine Trouble and Its Treatment

**Published:** 1878-07

**Authors:** 


ATLANTA
Medical and Surgical Journal.

Vol. XVI.] JULY—1878. [No. 4

### Original Communications.

### UTERINE TROUBLE AND ITS TREATMENT.

By A COUNTRY M.D.

Endometritis, whether existing as a result of subinvolution, malformation, malposition, or what not, is always, in its chronic form, more or less obstinate, and requires tedious treatment for its complete cure.

That the general system should be deleteriously impressed by the uterine disturbance set up by this inflammation, is inevitable, and not to be wondered at, since the sympathetic connections existing between the uterus and all other vital organs is so intimate, that uterine disturbance of almost any nature, or of any duration, exhibits a qualified disturbance in remote parts. This reflex derangement constitutes not, however, the sum of evils usually met with in a case of chronic endometritis. Local difficulties exist which, though not so extensively harrassing as the reflex, yet are sources of considerable annoyance, discomfort, and even at times, serious complications. But a graphic delineation of the pathological diversities or peculiarities coincident with this complaint is not necessary; every physician is acquainted with them. They, collectively and severally, serve to form the pathognomonic symptoms of uterine derangement, and in connection with the various modes of local examination, designate the individual character of the malady. One ridiculous and unwarranted phase exists in uterine practice, and is referable to the want of intelligence or want of information with the laity. In certain locations, (it may be remote or it may be contiguous to populous centres, where professional talent and scientific requirements concentrate and control ignorant prejudice), there exists a characteristic antipathy against all forms of local uterine treatment. Persons affected with this prejudice tenaciously cling to the belief that the temporary relief afforded by the various lenitive remedies is either sufficient, or as much as can be accomplished toward a cure. In instances where they may be induced to a contrary view, they submit with a lingering reluctance.

Great difficulty is experienced at times in inducing to this course; and particularly is this embarrassment felt by the younger members of the profession, and *more particularly* by those unfortunate beings embraced in the category of "*unmarried young doctors.*" What is the talismanic influence that impels this prejudice—that instigates this unjust predeliction for "old married men" in duties where the young would prove equally as efficient? It is not a consideration of competency, but of social predisposition. It is not, certainly, a question of propriety, but it must be an unjust impression of moral delinquency.

On account of these various reasons, the majority of cases of this class are of the chronic kind, and will, of necessity, have to be subjected to constant and protracted treatment before a cure can be realized.

The measures to be adopted in the treatment are of twofold character, viz: constitutional and local. It is invariably the case that the reflex nervous derangement has fully established the so-called hysterical condition, with its concomitant features, anæmia and general debilty. Pain, aches and functional derangement of various organs exist as manifestations of cerebral or spinal disturbance. Remedies whose action tends to obstruct the reflex activity of the spinal centre are here indicated, and the bromides and chloral will be found to be our best agents. Spinal stimulants, too, are here very serviceable; and amber, asafœtida, etc., may well be combined with the foregoing.

To change the depraved blood condition is a very important indication, since the improved nervous state could not be long sustained with a blood dyscrasia. Here the preparations of iron, in combination with those drugs that benefit the digestion, are essential, and will result in much good if assiduously administered. Hot hip baths and hot foot baths will be found no insignificant auxiliaries, and should always be resorted to.

Local measures consist of those that are medicinal and mechanical.

For the local application of medicines, we have as the appliances most worthy of note, Simpson's uterine syringe, and Taliaferro's uterine tent. The latter, as combining the component parts of the local treatment, (medicinal and mechanical) is superior to every other instrument. The tent is saturated with the alterative, astringent or cathartic, and introduced into the uterine cavity, where it comes in contact with the whole diseased surface, fundus and cervix. Independent of any direct or elective action of the medicines, the tent exerts its own benefit—which is stimulant and tonic—to the displaced or weakened uterus.

The principal articles used for this local application are iodine, carbolic acid, and tinct. ferri chloridi. The idea advanced by some physicians, more ideal than practical, that an injection, full strength, of tinct. ferri into the vagina alone, until a cast of the cavity has been thrown off, as a result of the cauterization, can claim no merit except of being barbarous. A weak injection of this tincture, however, is very valuable in vaginal leucorrhœa; yet it serves no advantage to the uterus, not even on the theory of counter-irritation.

I have just seen a case in which a contracted cervix and os have given rise to a series of hysterical phenomena, as well as a chronic endometritis, that well illustrates the importance of immediate local treatment. The subject, a maiden, was formerly subject to epileptiform convulsions, but probably the thorough establishment of the catamenia was the cause of the modification. She has no fits now, but most distressing headaches. I propose using a sponge tent on this case, and hope to be able to report to you a favorable result.

